# Dementia in primary care: a qualitative study with general practitioners and caregivers with and without migration backgrounds

**DOI:** 10.1186/s12875-025-02952-5

**Published:** 2025-09-16

**Authors:** Nele Kornder, Nicole Lindner, Meinert Ehm, Felix Rachor, Cheng Wieli Shan, Stefan Bösner, Veronika van der Wardt

**Affiliations:** Department of Primary Care, Philipps-Universität Marburg, Karl-Von-Frisch-Straße 4, Marburg, 35043 Germany

**Keywords:** Dementia, General Practitioners, Caregivers, Delivery of Health Care, Transients and Migrants

## Abstract

**Background:**

Dementia care presents significant challenges for caregivers and healthcare providers. Understanding these challenges, particularly in the context of cultural diversity, is essential for improving care quality. This study examines caregiving experiences among three groups—caregivers without migration background, caregivers with migration background, and general practitioners (GPs)—to gain insights into their distinct challenges and identify areas for support.

**Methods:**

An explorative qualitative study design was used, employing semi-structured interviews with participants from three groups: caregivers without migration background, caregivers with migration background, and GPs. Participants were recruited in Hesse, western-central Germany, through GP practices, dementia support organizations, and social institutions. GPs were drawn from the research network of the Department of Primary Care in Marburg. Interviews, conducted between October 2020 and June 2021, were analyzed thematically. Findings were compared across groups to identify shared and unique experiences.

**Results:**

The analysis revealed six main themes: 'Core health care challenges,' 'communication approaches and information sharing,' 'role of GP,' 'situation of caregivers,' 'influence on caregiver-GP relationship,' and 'socio-cultural barriers and care preferences'. Key differences emerged in GP approaches to dementia diagnosis and communication, as well as in expectations of the GP's role. Caregivers expressed the need for more time, empathy, and information from GPs, while GPs highlighted challenges such as limited consultation time and concerns about treatment effectiveness. Caregivers with a migration background experienced additional challenges such as cultural and linguistic barriers, which impacted their access to and navigation of dementia care.

**Conclusions:**

This is the first qualitative study to explore caregiving experiences across these three groups. The findings highlight the importance of addressing the specific needs of dementia patients and their caregivers, particularly those from migration backgrounds. Enhancing culturally sensitive support services and promoting cultural competence among healthcare providers are critical to improving dementia care. Addressing these gaps requires collaborative efforts to better align healthcare services with the diverse needs of caregivers and patients.

**Supplementary Information:**

The online version contains supplementary material available at 10.1186/s12875-025-02952-5.

## Background

Dementia is the seventh leading cause of death in older people worldwide, in Germany it is the third [1]. There are an estimated 65 million people living worldwide with dementia now, which is likely to double by 2050 [2]. In Germany alone, the number was estimated around 1.8 million in 2021 [3]. Dementia is a chronic, progressive syndrome, characterised by the acquired loss of two or more cognitive abilities by brain disease or injury [4].

Management typically begins in primary care, where general practitioners (GPs) play a key role [4]. In Germany, nearly all GPs report treating patients with dementia [5]. However, adherence to clinical guidelines [6, 7] in dementia care varies: While most GPs follow core recommendations such as medication reviews and physical examinations, other aspects—such as palliative care discussions, diagnostic imaging, and non-pharmacological interventions—are less consistently implemented [8, 9]. GPs often express skepticism toward dementia screening tools and concerns about the emotional impact of diagnosis, despite recognizing their pivotal role in early detection and disease management [10, 11].

Family caregivers are essential in supporting people with dementia [12]. However, in Germany, almost three quarters of caregivers of them have at least one unmet need regarding the person they care for indicating the pressure they experience [13]. These needs often relate to safety, physical and mental health of the care recipient, but also significantly concern the caregivers’ own wellbeing [13–15].

For caregivers with a migration background, these challenges can be more complex [16, 17]. Germany’s population is increasingly diverse, with around 21.2 million people—25.6% of the total population in 2024—having a migration background [18]. Common countries of origin include Turkey, Poland, Russia, Kazakhstan, Rumania and Syria [19]. As this population ages, age-related illnesses such as dementia become more relevant. Older migrants and their families often face structural barriers to accessing dementia care, such as language difficulties, stigma, and a lack of culturally sensitive services [20–22]. These barriers can be particularly pronounced in rural areas, where healthcare infrastructure is often limited. Policies such as the 2016 residence obligation have further increased the number of migrants in less urbanized regions, where long distances, limited public transport, and scarce interpreter services complicate access to care [23].

In 2020, the German government launched the National Dementia Strategy [7], which outlines general measures to support family caregivers, including targeted initiatives for those with a migration background. However, this population has so far received only limited attention within both policy implementation and research. The strategy’s real-world impact, especially in rural regions, remains insufficiently understood—particularly concerning the lived experiences of migrant families.

These challenges are not unique to Germany. Similar patterns have been documented in other high-income countries, where delayed diagnoses, insufficient culturally tailored services, and inadequate support for informal caregivers—especially among migrant and minority groups—persist in primary care settings [24–26].

This study aims to explore the following research questions:

What are the experiences and expectations of caregivers for people with dementia, including those with a migration background?

What are the perspectives of GPs on the care of dementia patients and their caregivers?

## Methods

### Design

We conducted a qualitative study exploring the experiences and expectations regarding dementia care in Germany among three participant groups:

1. Caregivers without migration background,
2. Caregivers with migration background, and
3. GPs

The three data sets were collected as separate but thematically related studies, each focusing on a distinct stakeholder group. Participants were not recruited as matched pairs to allow open expression and reduce social desirability bias. Preliminary analyses revealed overlapping themes, prompting integration of the findings to enable a more comprehensive and nuanced understanding of dementia care and communication dynamics.

The study was guided by a reflexive thematic analysis approach, emphasizing the researchers’ active role in interpreting the data. Reflexivity was ensured through regular discussions to reflect on assumptions and perspectives within the team.

### Patient and public involvement

We presented the project and preliminary findings to a Primary Care Patient and Public Involvement (PPI) group to explore whether the emerging themes resonated with their experiences. While this input was not used as a formal validation or member-checking procedure, the exchange provided informal reflections that supported the relevance of the findings. The PPI group consisted of individuals with regular contact to primary care settings with some of them having care experience.

### Participants

The study included two participant groups (group 1 and 2) of family caregivers and one of GPs (group 3). Caregivers were eligible if they accompanied a relative with any type of dementia regularly to GP visits within the last 12 months, had sufficient German language skills to participate in the interview, and were able to provide informed consent. Individuals with a diagnosis of dementia themselves were excluded. One caregiver group included participants with a migration background from a non-German-speaking country (first or second generation) (group 2). The GP group (group 3) consisted of practicing GPs based in the state of Hesse (Germany).

### Recruitment

#### Recruitment of caregivers

Participants for both studies with caregivers were recruited in GP practices, organisations supporting people with dementia and their caregivers (e.g. the German Alzheimer’s Society) and other institutions from the social or charitable sector (e.g. geriatric psychiatric counselling). For the study including caregivers with a migration background, we also contacted organisations providing support for people with migration backgrounds (e.g. the Turkish Alzheimer's Society). The participants were recruited by informing the coordinators of the organizations and by distributing materials such as posters and flyers within the organizations. We used purposive sampling to include participants who could provide relevant insights. GP practices and support organisations were first contacted by telephone and then sent study information for potential participants. The study was explained and eligibility criteria were checked when candidate participants contacted the study team. All participants provided written informed consent prior to participation. No compensation for expense was paid.

#### Recruitment of GPs

We conducted an Internet search to identify potential GPs working in the state of Hesse. We then contacted each GP by phone, and those who expressed interest received study details and an interview was scheduled. Written informed consent was obtained from all participating GPs, and no compensation for expense was provided.

### Interviews

The interview guides were developed based on a review of existing literature on informal caregiving for people with dementia and healthcare access, particularly in relation to migration and diversity, to ensure relevance and comprehensiveness. In addition to academic sources, the guides were informed by practical considerations and professional experiences of the research team. The key topics on which the guide was based are presented in Table 1. Given the exploratory nature of the study, the interview guides were intentionally designed in a semi-structured and open-ended format to allow participants to raise a wide range of experiences beyond the predefined topics. After conducting the first interviews, the research team discussed emerging findings and refined the guides iteratively to better capture relevant themes. The sample questions covered in the semi-structured guides are outlined in Table 1. Additional demographic data was collected through a brief accompanying questionnaire (see Table 1 for an overview of questions).

**Table 1:**
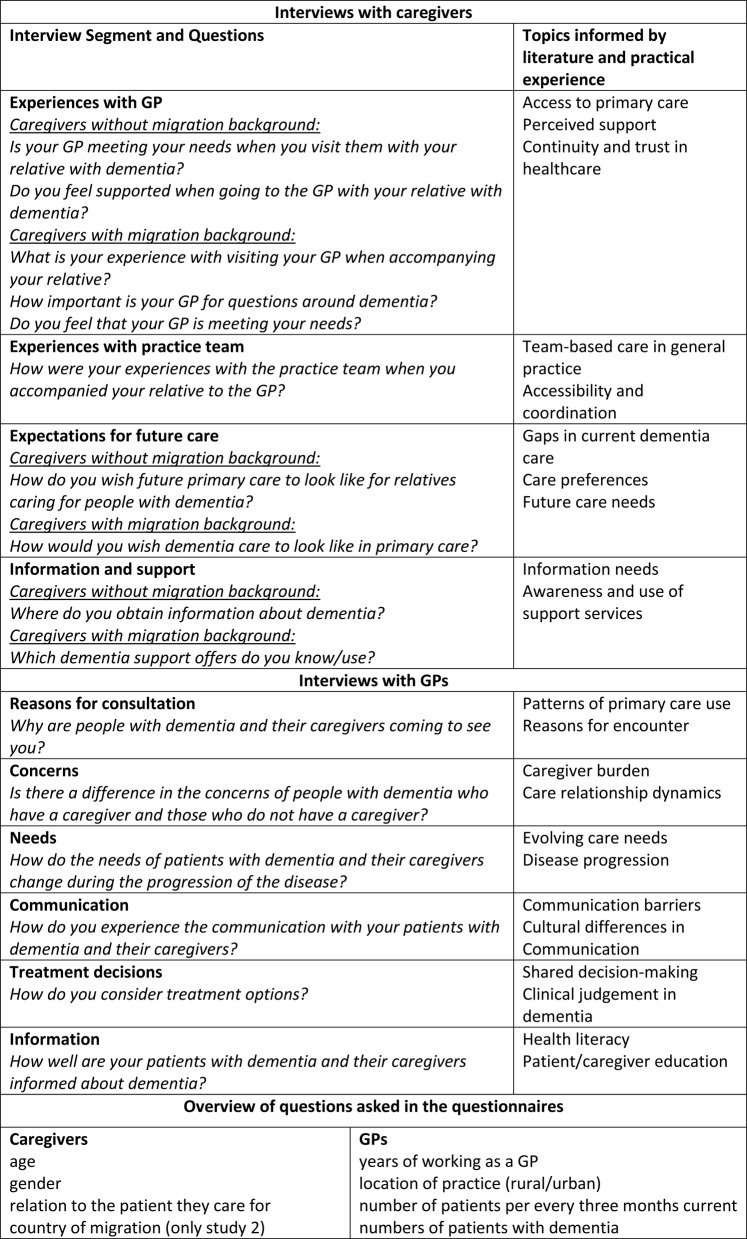
Topics and example questions of the interview guides and overview of questions asked in the questionnaires

The full interview guides, including the accompanying short questionnaires for each study, are provided as Supplementary Materials 1–3.

The interviewers were three male medical students (study 1: ME, study 2: CWE, study 3: FR), each working on a project towards their doctoral degree. They received training in qualitative interviewing from VvdW, an experienced female researcher with a background in psychology and dementia research, who also provided ongoing feedback throughout the study. Interviews were conducted either by telephone or in person (at participants' homes or surgeries). The interviews were carried out between October 2020 and June 2021 and took between 17 and 108 min.

### Analysis

Audio recordings of all interviews were transcribed verbatim by the respective interviewer (CWS, ME, FR). A reflexive thematic analysis was conducted following Braun and Clarke [27, 28]. The analysis combined both inductive and deductive strategies: existing literature and prior assumptions informed the initial coding framework (deductive), while themes were also allowed to emerge directly from the data (inductive). This approach was informed by both the interview guide and the underlying evidence base, while remaining open to insights emerging directly from the data.

First, each interviewer (CWS, ME, FR) conducted an initial thematic analysis of their interviews, including transcript review, coding, and reflective memo writing. Second, VvdW reanalyzed all interviews, synthesizing data across cases by reviewing memos, discussing interpretations with the team, and comparing themes across participant groups.

The study team is multidisciplinary: VvdW has a background in psychology and health research in dementia and is highly experienced in qualitative research, SB is an experienced GP and researcher, CWS, ME, FR are medical students and NL, NK are academic and practising GPs with experience in qualitative research.

The composition of the study team enriched the analysis by bringing diverse perspectives. Clinical researchers contributed practice-based insights, while the psychological perspective emphasized emotional and communicative aspects. Medical students challenged assumptions, and VvdW ensured methodological rigor through her qualitative expertise and guidance in reflexive discussions. In addition to disciplinary diversity, we also considered gender aspects in our team composition and reporting. The interviewers were male medical students, while the qualitative trainer (VvdW) was a woman. While we did not observe systematic effects of gender on the interview dynamics, we decided to report this information to support transparency and reflexivity.

The study process underwent an internal peer review by the entire working group and preliminary results were presented at the German GP conference.

Interviews were conducted in German, with quotes translated for publication. Data management and analysis were supported by MaxQDA (2022) and Microsoft Excel (2018). ChatGPT (OpenAI, GPT-4) was used to support the language editing and refinement of this manuscript; all outputs were critically reviewed and approved by the authors to ensure accuracy and integrity.

Data saturation was considered reached when no new themes emerged in consecutive interviews, as determined jointly by VvdW and the respective interviewer through comparison with the established codebook.

## Results

### Participants

In total, 38 caregivers participated in this study, including 20 caregivers without a migration background and 18 caregivers with a migration background. Additionally, 17 GPs were included. Participant characteristics are detailed in Tables 2, 3 and 4.

**Table 2:** Caregivers without migration backgrounds

ID code	Age Range (in years)	Gender	Relation to person with dementia
CG_01	30–34	man	Parent
CG_02	70–74	woman	Partner
CG_03	70–74	woman	Partner
CG_04	55–59	woman	Partner
CG_05	80–84	woman	Partner
CG_06	85–89	woman	Partner
CG_07	40–44	woman	Parent
CG_08	70–74	man	Partner
CG_09	55–69	woman	Partner
CG_10	50–54	woman	Parent
CG_11	75–79	woman	Partner
CG_12	65–69	woman	Partner
CG_13	70–74	woman	Partner
CG_14	50–54	woman	Both parents
CG_15	75–79	man	Partner
CG_16	80–84	woman	Partner
CG_17	85–89	man	Partner
CG_18	80–84	woman	Partner
CG_19	75–79	man	Partner
CG_20	60–64	man	Parent

**Table 3:** Caregivers with migration backgrounds

ID code	Age Range (in years)	Gender	Country	Generation	Years lived in Germany	Relation to person with dementia
CGM_01	60–64	woman	Türkiye	1	48	Parent
CGM_02	50–54	woman	Croatia	2	53	Parent
CGM_03	75–79	woman	Czech Republic	1	51	Partner
CGM_04	55–59	woman	Kazakhstan	1	30	Parent
CGM_05	45–49	woman	Türkiye	2	47	Parent
CGM_06	65–69	woman	South Korea	1	45	Partner
CGM_07	65–69	woman	Iran	1	43	Partner
CGM_08	45–49	woman	Türkiye	2	46	Parent
CGM_09	45–49	woman	Sinti	2	48	Parent
CGM_10	75–79	woman	Portugal	1	49	Partner
CGM_11	55–59	woman	Kazakhstan	1	30	Mother-in-law
CGM_12	35–39	woman	Syria	1	3.5	Parent
CGM_13	55–59	man	Türkiye	1	41	Parent
CGM_14	85–89	man	Ukraine	1	20	Partner
CGM_15	35–39	woman	Türkiye	2	39	Parent
CGM_16	65–69	woman	Bulgaria	1	30	Parent
CGM_17	50–54	woman	Croatia	2	51	Parent
CGM_18	35–39	woman	Türkiye	2	39	Parent

**Table 4:** GP participants

ID code	Gender	Years of working as GP	Location of practice	Number of patients per 3 months	Number of patients with dementia currently
GP_01	man	< 10	Rural	> 1500	> 25
GP_02	woman	10–20	Urban	> 1500	> 25
GP_03	woman	> 20	Urban	1000–1500	10–25
GP_04	woman	10–20	Urban	< 1000	< 10
GP_05	woman	> 20	Urban	> 1500	> 25
GP_06	man	> 20	Rural	> 1500	> 25
GP_07	woman	< 10	Urban	1000–1500	10–25
GP_08	man	10–20	Urban	< 1000	10–25
GP_09	man	> 20	Urban	1000–1500	> 25
GP_10	woman	> 20	Rural	> 1500	10–25
GP_11	man	> 20	Rural	> 1500	> 25
GP_12	woman	> 20	Rural	1000–1500	> 25
GP_13	man	> 20	Urban	unknown	10–25
GP_14	woman	< 10	Urban	< 1000	> 25
GP_15	man	> 20	Urban	< 1000	10–25
GP_16	woman	> 20	Urban	1000–1500	> 25
GP_17	man	> 20	Urban	1000–1500	10–25

The age of caregivers ranged from 31 to 87 years. Of these caregivers, 20 were spouses of the person they cared for, while 17 were children and one a daughter-in-law. Among caregivers without migration background 14 were women and among the caregivers with a migration background 16 were women. The caregivers with a migration background originated from ten different countries.

The sample including GPs was well balanced in terms of gender (9 female, 8 male), numbers of patients per three months and numbers of dementia patients. 11 GPs had more than 20 years of working experience as GP and 12 GPs worked in urban areas.

### Thematic findings

Thematic analysis revealed a broad range of experiences and perspectives related to dementia care in general practice. Across all participant group, the data reflected recurring patterns in how dementia is diagnosed, communicated, and managed, as well as the in the roles and expectations placed on caregivers and GPs. The six overreaching themes are:

1. Core challenges in health care
2. Communication approaches and information sharing
3. Role of GP
4. Situation of caregiver
5. Influence on caregiver—GP relationship
6. Socio-cultural barriers and care preferences

**Fig. 1:**
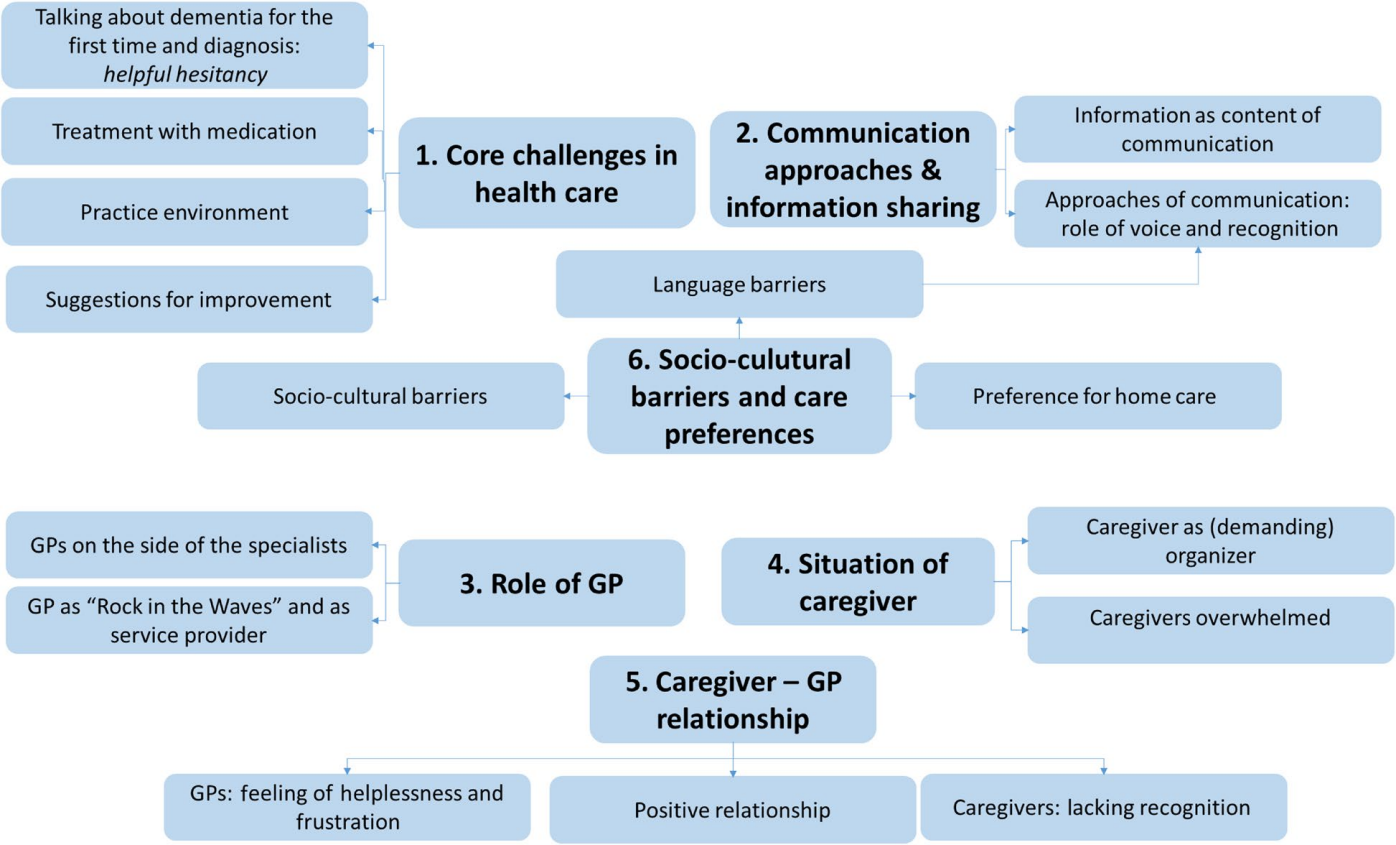
Graphical presentation of the main themes and subthemes derived from the interviews

Please see Fig 1 for a graphical presentation of the main themes and sub-themes

### Core challenges in health care

#### Talking about dementia the first time and diagnosis

GPs varied in their approaches to discussing dementia, with some referring to specialists immediately and others delaying action until symptoms progressed:

*“When they come the first time [with suspected dementia], I wait, because it’s a diagnosis that is very limiting for the patient. And then I observe, three months, half a year, a year to see if it’s fast progression, or slow, and then with more help and offers, what you have to do, then also with more communication.” (GP_12)*

This hesitancy may have stemmed from an awareness of the emotional and social consequences of a dementia diagnosis. Rather than acting immediately, some GPs adopted a cautious approach to protect the patient’s sense of autonomy and avoid potential stigma. Such “protective delay” may, however, lead to postponed care planning.

In our sample, GPs overwhelmingly reported that it was mostly relatives who wanted the person they care for to be diagnosed, often due to behavioral issues or sleep problems. Only when the person living with dementia was in the very early stages did they come to see the GP.

*“Usually, it’s the relatives who come. I would say that there is a difference. Patients who have a very mild cognitive impairment, they often come themselves and say ‘Yes, I realised, I am a bit forgetful, what can I do about that? Do I have to do something? Can anything be done?’ For patients who progressed a bit further, relatives often approach us first.”* (GP_10)

Approaches to diagnosis varied: while some GPs relied on cognitive tests and imaging, others were more reluctant to refer—citing lack of effective treatment or concern about the psychological burden of a formal diagnosis.

*“The only problem was that the doctor didn't realise that my husband actually had dementia. I found that problematic. I can then take everything else into my own hands.” (CG_05)*

Some GPs used tests (e.g., Dementia Detection Test [DemTect], Mini-Mental State Examination [MMSE], clock test). Others would also perform blood tests and imaging before referring. However, not all GPs prefer referrals, doing so only if necessary or requested by patient or caregiver.

GPs’ reluctance to refer to specialists was often due to the perceived lack of effective treatment. They also viewed the diagnosis as disruptive for the patient and caregiver, creating fear and potentially stigmatizing the person with dementia:

*“…when you suggest ‘go do a dementia support group with your caregiver, get advice from [support organisations]’, then they are afraid of the stamp on their forehead [i.e., stigmatisation] …Yes, the fears are still very, very strong.’ (GP_12)*

These patterns suggest a deeper tension: GPs navigate between professional caution and patients' and caregivers’ needs for clarity and action. This "helpful hesitancy" reflects a balancing act between harm avoidance and timely support.

#### Treatment with medication

Medication decisions further reflected this balance. GPs described prescribing as a response to caregiver expectations rather than medical necessity:

*“If the urgent need for placebo-treatment with ginkgo biloba is desired, and their salvation depends on it, then I will prescribe.” (GP_06)*

While some saw value in antidementia drugs, most were skeptical. Medication served not just therapeutic purposes but also symbolic ones—signaling care, responsiveness, and support. Discontinuation, on the other hand, could be perceived as withdrawal of care:

*“… if you tell them, they have to stop [the medication], they completely despair.” (GP_15)*

This indicates that prescribing practices were shaped as much by relational dynamics and caregiver reassurance as by clinical indication.

#### Practice environment

The practice environment was generally viewed as friendly and supportive by the caregivers. Nevertheless, participants described how visits to the GP practice were complicated by both structural factors and dementia-related symptoms.

*“I can’t go to the GP with him. I insist on home visits, it just does not work anymore. I can’t go there with my husband. It takes too long and then he starts in the waiting room and wants to move things around.” (CG_04).*

#### Suggestions for improvement

Both caregivers and GPs had suggestions for improving health care for people living with dementia. Many caregivers wished for more time to talk to the GP but also for more empathy, better listening and a more open attitude:

*“Something was missing. They should not only listen, prescribe and send you away. I think they should approach patients more. Also, a bit more time. I don't think it's important for a doctor to have more patients, but rather fewer patients as far as I'm concerned. But more time for patients.” (CGM_08)*

GPs, meanwhile, suggested expanding community-based services and non-commercial informational materials.

### Communication approaches and information sharing

#### Approaches of communication

GPs had different views and experiences on how to communicate with people with dementia and caregivers. Several GPs would find communication with the person with dementia difficult. For some, the time the consultations would take was an issue, others did not want to listen to the confusion with which some patients communicated. One GP expressed almost a rejection of communicating with patients:

*“I don’t want to listen to the stupid prattle, whatever he is imagining […] in his fantasy. That’s no fun.”* (GP_17)

Such statements may reflect more than just challenges in managing communication—they can also point to underlying difficulties in maintaining the voice and recognition of personhood in the context of dementia care. When the communicative contributions of a person with dementia are perceived as confusing or irrelevant, it may limit their opportunities to participate actively in conversations. These situations illustrate how dementia can affect not only cognitive abilities but also the ways in which others engage with the person—either as a dialogue partner or primarily as a care recipient.

Depending on the patient’s cognitive abilities, other GPs regarded the communication with the person with dementia as generally going well, particularly if they knew them well and they had sufficient cognitive abilities to come to the practice.

There were also differences between GPs in their focus of communication. Some would mostly communicate with the caregiver; others would focus on the person with dementia:

*“I always talk to the person with dementia and not with the caregiver sitting next to the patient about him. Yes, and then I see how he reacts, what he wants to know, what he doesn’t want to know, does he ask me something.”* (GP_17)

This quote illustrates an ideal of relational respect: the GP seeks not only to include the person with dementia but to attune to what information they want, thus affirming their agency. However, this was not consistent across practices. In some cases, communication also occurred between care services and GPs without including either the person with dementia or the caregiver—for example, when medication changes were discussed.

For caregivers, the GP could act as a communication bridge—helping to clarify difficult conversations or validate caregiving efforts. Not all caregivers preferred joint visits with the person they cared for; some valued the privacy of separate consultations, where they could "*talk more openly*" (CG_17). This highlights how the caregiving relationship itself may shape expectations around whose voice should be prioritised, and under what circumstances.

#### Information as content of communication

Receiving information was important for caregivers but not always provided by the GP:

*“You don't get any information [about dementia] there [at the GP].”* (CG_04).

Most caregivers would turn to other sources like the Alzheimer Society, Internet, and relatives who have experience in caring for someone with dementia. As outlined above receiving more information from the GP was identified as a patient need.

On the other hand, GPs perceived caregivers as quite knowledgeable or “*well informed*” (GP_08). One GP was reluctant to discuss the progression of dementia with the caregivers:

*“[…] describing the course of dementia to relatives in order to prepare them for what they might have to expect [...] is not particularly useful because, firstly, it progresses very differently, secondly, the time frame is very different and, thirdly, since the majority of people involved are over eighty, a variety of potentially serious illnesses can occur that make dementia seem insignificant in the context of life organisation, heart attacks and carcinomas and who knows what else.”* (GP_06)

This perspective reflects uncertainty around prognosis and a specific view of medical relevance. It may overlook the symbolic and emotional value of information, which helps caregivers and patients prepare for the future and maintain a sense of control. The way information is shared—or withheld—can signal whether their needs and experiences are acknowledged.

### Role of GP

#### GPs on the side of the specialists

Caregivers perceived the GP as important for the care and as being supportive but competency for dementia was attributed to specialists:

*“Yes, so as I said, he* [the GP] *won't have this competence for dementia. But he can say: ‘I can help until then. And at that point, the specialist, i.e. the neurologist, has to get involved.’"* (CG_15)

GP’s views reflected this to some extent, as they considered signposting to specialists to be part of their role. But the role extended beyond this: one GP described himself as *“a bit of a counsellor”* (GP_04).

Many GPs saw themselves as responsible for the "overall care of the family". Not just in a purely medical context, e.g. organising professional care, mediating family disagreements and sometimes legal help. Some stated that they "always had an open ear" for the family. Although, as outlined above, some GPs completed diagnostic and prescribed medication, most GPs considered monitoring additional health issues more within their remit. They saw themselves as a kind of "gatekeeper"/ "guide" who is the first point of contact, helps with the organization of care and monitors comorbidities.

*“Well, let's start with psychology. That means being available as a contact person for whatever things, purely technical, care organization and ultimately also medical monitoring of the other deficits that are there due to/ caused by heart disease, diabetes, etc., and advising the relatives and I think that's the most important thing. Because often all the bureaucratic, uh, things are not even manageable for many people, care organization, how does it work, applying for care, what about a living will, what about a care directive and, and, and, and. So that's what I do.”* (GP_06)

#### GP as service provider—GP as “rock in the storm”

For some caregivers, trust and empathy were important aspects of their relationships to the GP, others considered signposting as the main task of GP for the healthcare of people with dementia:

*“Just like with all other illnesses, you go to the GP just to see which doctor you should go to. And get a, I don't know, referral.”* (CGM_07)

Additionally, GPs had different perspectives on their relationships with the patients. For some it was providing a service, others felt they were a contact person for the caregivers and patients, and tried to make them feel save:

*“Yes, give the patient a certain sense of safety. Give him the feeling that someone is looking after me.”* (GP_17)

### Situation of caregiver

The theme "Role of Caregiver" was primarily discussed by GPs, while for caregivers, it was crucial to be acknowledged and asked about their own well-being:

*“Even when he has spoken to my husband and treated him, he never forgets to ask how I am.”* (CG_16)

"Role of Caregiver" included the sub-themes "caregiver as (demanding) organizer" and "caregivers overwhelmed".

#### Caregiver as (demanding) organizer

GPs saw the caregiver as the person who would organise GP visits and healthcare for the person with dementia. Some GPs perceived caregivers as rather demanding:

*“And the patients themselves, they are, as I said, um, less demanding, yes, they are actually rather reserved, they bring it up when they notice it, but they don't actually want to have it clarified. On the other hand, the caregivers, they're very much pushing because they're afraid that it will get worse and they don't know how to deal with it, yes. That's what strikes me.”* (GP_04)

#### Caregivers overwhelmed

GPs were aware that caring for a person with dementia can be overwhelming for the relative, in particular if there is no one to share responsibilities with as caring includes a lot of bureaucracy. GPs recognising this, pointed the caregivers in the right direction:

*“You also have to reach out to them and tell them "You apply for the care degree, the incontinence materials, take care of the severely disabled person's pass". You need the degree of care and severe disability for taxi licences, for trips to the doctor if the family is not available. So, there's a lot of organisation to do.”* (GP_12)

### Influence on caregiver—GP relationship

#### Positive relationship

Most but not all caregivers felt positive about their relationship with their GP. Some emphasised that they felt not only supported in their care for the person with dementia but also themselves in their role as caregiver.

*"[My GP is] very important because if I don't know what I'm doing, I call him.” (CGM_16)*

#### Caregivers: Lacking recognition

However, some caregivers missed that aspect:

*“My [needs]? No. No, because I'm not the patient for him. […] That I'm always asked how my husband is and that he NEVER asks me how I am. And he said: ‘Then come to me as a patient and I'll ask you that.’”* (CGM_16)

Moreover, certain caregivers felt that the GP is not listening to their needs, or felt “*insecure” (CG_13)* because the GP could not remember them from their last visit.

#### GPs: Feeling of helplessness and frustration

GPs sometimes expressed feeling helpless in relation to both the patient and the caregiver. This sense of helplessness often led to the search for extreme solutions:

*“Because then you almost wish that they would somehow fall, break something, come to the clinic (…) and then a therapy is initiated” (GP_15)*

In some cases, the feeling of helplessness led to a defensive attitude and patronising talk about the patient:

*“I often have to deal with poor, weak, helpless, underprivileged, unintelligent, simple people. That’s annoying.” (GP_17)*

The GPs also described an "*over-engagement*” (GP_06) of relatives. Some carers would not personally participate in care, but would instead burden people who are involved in care with their demands.

*“The THIRD daughter in turn writes NEW e-mails about what to do with the 89-year-old lady and so it goes on”” (GP_06)*

### Socio-cultural barriers and care preferences

#### Socio-cultural barriers

Overall, socio-cultural barriers were considered important (especially language barriers, inexperience with the healthcare system) and a need for culturally specific help (lack of culturally suitable care homes, culturally sensitive help services) was seen. In particular, unfamiliarity with the German healthcare system could lead to "*stress and fear*" (CGM_12) why caregivers with a migration background saw themselves in a vulnerable position.

#### Language barrier

Caregivers with migration background highlighted the presence of a language barrier, which they saw as a critical factor shaping their interactions. This barrier was particularly significant as effective communication was considered as essential for contact with their GP.

This issue was especially relevant for older people who had learned German as a foreign language and were more prone to forgetting it as dementia progressed. For most participants, language barriers were thus associated with both dementia and migration background. Family or professional help was often needed to translate:

*“Well, my fear or the thought is: if his language skills deteriorate even further—he's already becoming, you know, much quieter. And he talks less and also can’t follow longer conversations as well anymore. Right. And in situations where he used to get very upset, he would angrily swear in Croatian.”* (CGM_17)

This quote illustrates not only the communicative challenges but also deeper concerns regarding loss of voice and personhood**.** As dementia and language loss converge, caregivers feared that their relatives with dementia might no longer be “understood,” both literally and existentially.

#### Preference for home care

Caregivers with a migration background usually preferred home care, stating three main reasons: (1) family care was better or more familiar, (2) lack of alternatives due to lack of services (e.g. language adapted), (3) it was considered (culturally) “normal” to take care of the family:

*"And that's then/ so you wouldn't do that, that you then/ so we wouldn't do that, that you then take them to a home where nobody speaks Turkish! There's just no culture, no music, no understanding. And that's a big, big shortcoming here in Germany" (CGM_05).*

Caregivers without migration background also saw residential care as the last option and preferred care at home. Nevertheless, the family often did not appear to be as available as for caregivers with migration backgrounds. The relatives (usually the children) often lived further away and were very involved in their work.

## Discussion

This comprehensive combined interview study with caregivers and GPs was conducted with the goal of understanding migration-specific concerns as well as the experiences and needs in dementia care within a primary care context. We synthesised the data into the six main themes 'core challenges in health care', 'communication approaches and information sharing', 'role of GP', 'situation of caregiver', 'influence on caregiver—GP relationship', 'socio-cultural barriers and care preferences' showing a wide range of views. The following discussion interprets these findings in the context of existing research and links to future research.

### Variability in dementia diagnosis approaches

GPs employed different strategies for diagnosing dementia. Some were reluctant to diagnose at all due to concern for the emotional reactions of patients and caregivers or doubts about the effectiveness of treatment. This form of cautious restraint could be interpreted as a kind of "*helpful hesitancy"*, where GPs aim to protect patients and families from distress. However, such hesitancy risks delaying diagnosis and limiting access to support. Iliffe et al. describe a related phenomenon as "*therapeutic nihilism"*—the belief that little can be done for people with dementia, which can lead to avoidance of diagnosis altogether [29]. In our sample, while some GPs delayed diagnostic clarification, others completed assessments in their own practice or waited until caregivers or patients actively sought a diagnosis. These patterns mirror previous research on variations in diagnostic approaches in German primary care [10, 30]. The emotional and ethical complexities surrounding dementia diagnosis continue to shape GP practices [31–33]. Unlike other studies, our interviews did not address whether patients have the right to know their diagnosis [32, 34].

### Communication challenges

Caregivers expressed a need for GPs to take more time, actively listen, and provide more information about dementia and available support. This need was not always met: while some GPs made a visible effort to include the person with dementia as an active participant in conversations, others expressed frustration or disengagement when faced with communicative challenges. Such reactions may reflect a sense of emotional overload but also a risk of marginalizing the person with dementia. As von Kutzleben et al. argue, insufficient communication can be perceived as disinterest—an interpretation echoed in caregiver accounts in our study [35]. Moreover, information was often withheld based on medical-logical considerations (e.g. variability of disease progression), while caregivers emphasized its symbolic and preparatory value. This highlights a gap in expectations between medical and relational communication. Interventions such as communication training and reflective practice may help GPs navigate this tension and foster more inclusive, person-centered conversations [36, 37].

### Support structures and comprehensive care models

GPs suggested new support structures such as specialised community workers and activity groups as well as information brochures. If GPs wanted to provide information, it was important that information material was not produced by a pharmaceutical company. While the German Alzheimer Society provides a range of different information formats as well as support groups, not all GPs were aware of this. However, many caregivers were able to find information from various sources including the Alzheimer Society, the internet as well as friends and family.

Despite often relying on specialists for treatment, GPs managed numerous requests from caregivers, some beyond traditional health care roles. A comprehensive care model, particularly for patients without caregivers, could be beneficial [38–40]. Primary care based case management approaches have shown to be the most effective model improving quality of life and caregiver burden [41]. However, such models have not yet been introduced nationwide in the German care system. Future research should explore the integration of case management approaches within the German healthcare system.

### Cultural sensitivity in care

Caregivers with a migration background shared similar experiences and expectations to caregivers without a migration background. Language barriers sometimes made direct communication with dementia patients challenging, highlighting the need for culturally sensitive support services to aid both caregivers and GPs in managing care effectively. Although the National Dementia Strategy [7] recognizes the need for culturally sensitive services, our findings show that these remain limited in practice, especially in the primary care setting. Similar barriers have also been reported in other European contexts—for example, a study from Denmark identified cultural and linguistic challenges as major obstacles in providing post-diagnostic dementia care to minority ethnic communities [42]. Future research should therefore investigate how culturally adapted training programs for healthcare providers can improve communication strategies and patient outcomes.

### Strengths and limitations

Our findings reflect diverse perspectives from GPs and caregivers with and without migration backgrounds, highlighting both shared and differing needs in dementia care. A key strength is the inclusion of underrepresented groups [43], particularly caregivers with migration backgrounds, allowing for comparative insights into their experiences.

However, the interview guides were not harmonised across participant groups, which led to some variation in the topics discussed. Interviewers were medical students with limited qualitative research experience, which may have influenced responses, though regular debriefings helped ensure contextual understanding.

The ethnicity of GPs was not recorded, though this could be a relevant factor in caregiver-GP dynamics. Due to Covid-19, many interviews were conducted by phone, which may have impacted conversational depth, although prior research suggests comparable data quality. The exclusion of non-German-speaking caregivers may have limited participation of those with greater language-related needs.

Finally, since we did not track how many GPs declined participation, we cannot assess potential non-response bias. Nonetheless, our recruitment strategy—based on an open internet search—helped to include GPs beyond academic or research settings, possibly reducing selection bias.

## Conclusions

Caregivers with and without a migration background reported similar needs, while GPs showed varying approaches to dementia care. This underscores the need for more structured support in primary care. Communication training for GPs—including culturally sensitive strategies and practical tools—could enhance confidence and improve consistency in care. Multilingual information and culturally attuned support services should be expanded and better integrated into general practice. Future research should evaluate how such interventions work in routine settings and assess their impact on families with migration backgrounds. Additionally, it would be valuable to explore barriers to implementing the culturally sensitive measures outlined in the National Dementia Strategy in everyday primary care.

## Supplementary Information

Supplementary Material 1.

Supplementary Material 2.

Supplementary Material 3.

## Data Availability

All data generated/analysed are available upon reasonable request with corresponding author.

## Abbreviation:

GP: General practitioner
PPI: Patient and Public Involvement
DemTect: Dementia Detection Test
MMSE: Mini-Mental State Examination
