# Supplementary material for: Dementia in primary care: a qualitative study with general practitioners and caregivers with and without migration backgrounds
Source: BMC Prim Care. 2025 Sep 16;26:282. doi: 10.1186/s12875-025-02952-5 (PMC12442260; doi:10.1186/s12875-025-02952-5)
Supplement: Supplementary file 1 — Supplementary Material 1. [file 12875_2025_2952_MOESM1_ESM.docx]

**Interview Guide: Caregivers without migration background**

Introduction

- Greeting and thanking the participant for taking part in the study
- Introducing yourself: The researcher briefly introduces themselves and their role in the study.
- Explanation of the project and the aim of the interview
- Participants are informed about the purpose of the project and the specific objectives of the interview.
- Consent form, explanation of voluntariness and data protection, completion of questionnaire. Participants sign the consent form if not already done, are reminded of their voluntary participation and data protection, and are asked to complete the short questionnaire
- Start of audio recording: Once all formalities are completed, the audio recorder is switched on to begin the interview.

Main part:

1. When was the last time you visited your GP?
2. Did your relative accompany you?
3. What is important to you when you go to your GP?

- What is the usual reason you visit your GP?
- What makes a good GP in your opinion?

1. How satisfied are you with your GP?

- What do you like or dislike about your GP?
- Why do you choose to go to this particular GP?
- How important is your GP’s opinion to you?

1. Does your GP know about your current situation?

- Does your GP come to your home to see your relative

1. Do you feel that your GP adequately addresses your needs?

- Does your GP take you seriously?
- Does your GP listen to you?
- Does your GP take enough time for you?
- Does your GP show understanding for your questions?
- Do you feel that after a conversation with your GP you have understood everything?

1. Do you feel supported by your GP?

- How does your GP specifically help you in dealing with your relative’s dementia?
- What does your relative’s current treatment with the GP look like?
- What is your relative’s current care situation?

1. What experiences have you had with the practice team?

- How do you perceive the support and organisation within the practice?
- Do you have to wait long for an appointment?
- What do you like or dislike about the practice?

1. What would you like from GPs in the future?

- What could your GP do better?
- What is already working well at the GP practice?
- Where or how do you inform yourself about memory problems or dementia?
- How important is your GP to you when you have questions about memory problems or dementia?
- Do you feel sufficiently informed?

1. What support services (in your area) do you know or use?

- How did you find out about them?
- What experiences have you had using these services?

Questions to Maintain the Conversation

- Can you think of an example for that?
- Do you remember a situation when
- Please tell me about it…
- You can take your time to think.
- What happened next?
- And then?
- How did it come about that…?
- You mentioned earlier that…
- Why…?
- What do you mean by that?
- Could you phrase that differently?
- Did I understand correctly that…?

Conclusion

Closing the interview

- "We are now coming to the end of the interview."

Final questions:

1. Is there anything else you would like to mention that is important to you?
2. Did we forget to ask anything?

"Thank you very much for your time and for taking part in the study!"

Give an outlook on what will happen next. Allow time for questions. Ask the participant if they would like to receive a summary of the study results after completion.

**Questionnaire (the questionnaires were administered as paper-based forms)**

1. How old are you? ____________
2. What is your gender?

Ο Female

Ο Male

Ο Other, namely: _______________

1. Which of the following best describes your place of residence?

Ο Rural community (< 5,000 inhabitants)

Ο Small town (5,000 – 20,000 inhabitants)

Ο Medium-sized town (20,000 – 100,000 inhabitants)

Ο Large city (> 100,000 inhabitants)

1. What is your relationship to the person you are supporting?

Ο Partner (spouse, life partner)

Ο Mother / Father

Ο Child

Ο Other relative (aunt/uncle, sister/brother, grandchild, etc.)

Ο Friend

Ο Neighbour

1. How often have you visited your GP in the past 12 months?

Ο Rarely

Ο Occasionally

Ο Often / Regularly
