# Supplementary material for: Dementia in primary care: a qualitative study with general practitioners and caregivers with and without migration backgrounds
Source: BMC Prim Care. 2025 Sep 16;26:282. doi: 10.1186/s12875-025-02952-5 (PMC12442260; doi:10.1186/s12875-025-02952-5)
Supplement: Supplementary file 2 — Supplementary Material 2. [file 12875_2025_2952_MOESM2_ESM.docx]

**Interview Guide: Caregivers with migration background**

Introduction:

- Greeting and thanking the participant for taking part in the study
- Explanation of the project (e.g. purpose and objectives of the questionnaire, interviews, etc.)
- Check consent, repeat information about voluntariness and data protection
- Fill out or discuss the questionnaire (for telephone interviews, send it beforehand)
- Introduction and start of audio recording:

Main part:

To repeat: This is about your experiences and expectations in general practice.

1. When was the last time dementia was addressed at your GP’s practice?
2. How is dementia addressed by your GP (diagnostics? therapy? counselling?)
3. How important is your GP to you as a contact person regarding dementia-related questions?
4. How does your GP support you in dealing with your relative’s dementia?
5. What would you wish for regarding dementia care in general practice?
6. Which (regional) support services for dementia care do you know, use, or find helpful?
7. How do you experience GP visits when you accompany your relative?
8. What do you think about GP visits? 🡪 What makes a good GP for you?
9. Do you take your doctor’s opinion seriously?
10. Do you feel taken seriously?
11. Do you feel that your GP considers your needs (during visits when you go together)?
12. How does caring for your relative affect your everyday life? -> How does your GP support you in caring for your relative? 🡪 How did the COVID-19 pandemic affect you?
13. How does your culture view dementia?
14. To what extent do you think your migration background or culture plays a role in your GP visits?
15. Do you think your migration background influences your communication? Does your GP speak your mother tongue (if it is not German)?
16. What are your experiences with the practice team or the practice itself?
17. What do you expect from the practice team or the practice itself?

Questions to maintain conversation:

- Can you think of an example for that? Please tell me about it…! What happened next? And then?
- Follow-up questions: I didn’t quite understand why…? Could you please explain/repeat that…? How did it come about that…? Regarding … you mentioned that…? Earlier you said that…? Why?

Conclusion:

- Closing the interview: We are now (almost) at the end of the interview.
- Final questions: Is there anything else you would like to add or mention? Did we forget anything?
- Thank the participant again.
- Give an outlook on what will happen next. Farewell.

**Questionnaire (the questionnaires were administered as paper-based forms)**

- How old are you:____
- Gender: m/f/d
- Migration background (country of origin or parents’ country of origin):
- Migration background in the ___ generation (please enter number).
- If not born in Germany: Approximately how many years have you been living in Germany?
- What is your (family) relationship to your relative with memory problems or dementia (e.g. son, daughter, husband/wife):
- How long have you been caring for your relative with memory problems or dementia (approx.)? ______________
