# Supplementary material for: Dementia in primary care: a qualitative study with general practitioners and caregivers with and without migration backgrounds
Source: BMC Prim Care. 2025 Sep 16;26:282. doi: 10.1186/s12875-025-02952-5 (PMC12442260; doi:10.1186/s12875-025-02952-5)
Supplement: Supplementary file 3 — Supplementary Material 3. [file 12875_2025_2952_MOESM3_ESM.docx]

**Interview Guide: GPs**

Introduction:

- Greeting and thanking the participant for their willingness to be interviewed:
- Informing the participant: Participants are once again informed about the qualitative interview study and data protection, and the framework conditions of the interview are finalised (duration, location).
- Signing the consent form and completing the short questionnaire: If not already done, the participant must sign the consent form and complete the short questionnaire. The researcher brings an additional copy of the study documents to each appointment to ensure the interview can be conducted without issues.
- Starting the recording: After all formalities have been completed, the recording device is switched on and the interview can begin.

Main part:

1. For what reasons are you (as a GP) consulted by people with dementia and their relatives?
2. What fears do people with dementia and their relatives have?
3. Are there different fears in people with dementia who come to the practice alone compared to those who come with relatives?
4. To what extent do the needs of your patients (people with dementia and their relatives) change over the course of the illness?
5. What organisational or non-medical problems do people with dementia and their relatives face?
6. How do people with dementia and their relatives react to the diagnosis?
7. How would you (as a GP) assess communication with your patients (people with dementia and their relatives)?
8. What therapy do your patients wish to receive from you (as a GP)? How are treatment decisions made?
9. How well informed are people with dementia and their relatives about the illness?
10. How good is your patients’ compliance?
11. What contribution can you (as a GP) make to meeting the unmet needs in healthcare provision for people with dementia and their caregiving relatives?
12. Is there anything that could support your work with people with dementia and their relatives? For example, specific informational materials for patients, group programmes (such as cognitive stimulation therapy), or additional support from community helpers to whom you could refer them?
13. Is there anything you would like to add that was not addressed in the interview but which you consider important?

Conclusion:

Thanking the participant for the interview: At the end of each interview, the recording device is switched off, and the researcher thanks the participant for the conversation.

**Questionnaire (the questionnaires were administered as paper-based forms)**

Sociodemographic Data

Gender

☐ Female

☐ Male

Specialization

☐ General Practitioner

☐ Internist

Professional Experience

☐ Less than 10 years

☐ 10–20 years

☐ More than 20 years

Practice Location

☐ Rural (fewer than 25,000 inhabitants)

☐ Urban (more than 25,000 inhabitants)

Type of Practice

☐ Solo practice

☐ Group practice

☐ Medical Care Center

☐ Other: _______________

Number of patients per quarter

☐ Less than 1,000

☐ 1,000–1,500

☐ More than 1,500

Number of dementia patients under treatment

☐ Less than 10

☐ 10–25

☐ More than 25
